# Emerging Risk Profile of Lung Cancer Therapy: Diffuse Alveolar Hemorrhage from Osimertinib

**DOI:** 10.1155/2019/6185943

**Published:** 2019-07-30

**Authors:** Michael J. Forte, Rahul G. Sangani

**Affiliations:** West Virginia University, Department of Pulmonary and Critical Care Medicine, PO BOX 9166 1 Medical Center Drive, Morgantown, WV 26506, USA

## Abstract

Osimertinib is an oral epithelial growth factor receptor tyrosine kinase inhibitor (EGFR-TKI) used primarily in the treatment of metastatic non-small cell lung cancer. It is usually well tolerated with less than 5% of patients developing significant pulmonary toxicity from the medication, typically within the first few months after initiation. Previously reported pulmonary adverse reactions include pneumonitis (nonspecific interstitial pneumonia or other forms of acute interstitial process), fleeting asymptomatic infiltrates on imaging, and eosinophilic pneumonia. We present an interesting case of a 65-year-old female with recurrent metastatic adenocarcinoma of the lung, treated with Osimertinib for 4 months, who developed a previously unreported toxicity of diffuse alveolar hemorrhage (DAH) requiring mechanical ventilatory support.

## 1. Introduction

Diffuse alveolar hemorrhage (DAH) is a potentially life-threatening disorder characterized by respiratory failure, hemoptysis, diffuse infiltrates on chest imaging, and blood loss anemia.

Prompt recognition and urgent evaluation with bronchoscopy are necessary for diagnosis. DAH is caused by a wide range of clinical entities. Common etiologies include capillaritis, vasculitis, and diffuse alveolar damage or from an extensive list of medications and toxins such as amiodarone, nitrofurantoin, cocaine, and tumor necrosis factor- (TNF-) alpha inhibitors [1]. To date, there have been no previously reported cases of Osimertinib causing DAH in the literature.

Osimertinib is an oral epithelial growth factor receptor tyrosine kinase inhibitor (EGFR-TKI) used primarily in the treatment of metastatic non-small cell lung cancer (NSCLC). It is routinely used in patients with metastatic T790M-positive NSCLC who have disease progression during or after EGFR-TKI therapy [2]. Osimertinib can now be prescribed as a first-line agent regardless of the presence of T790M. During early clinical trials [3], the most common pulmonary complication was interstitial lung disease, which occurred in about 4% of patients. In general, the drug is felt to have a favorable side effect profile and tolerability.

We present an interesting case of a previously unreported toxicity with the use of Osimertinib.

## 2. Case Presentation

The patient is a 65-year-old female who was previously diagnosed with moderately differentiated, invasive adenocarcinoma after a right upper lobectomy in 2013. The tumor was positive for EGFR mutation, KRAS wild type, ALK negative, CK7 positive, CK20 negative, and TTF negative. At that time, no chemotherapy or radiation was required. In 2014, the patient had a recurrence of her disease with a metastasis to mediastinal subcarinal lymph node. Over the next four years due to disease progression or inability to tolerate agents (e.g., development of acute pancreatitis from erlotinib), the patient was treated with multiple antineoplastic agents including bevacizumab, pemetrexed, durvalumab, tremelimumab, erlotinib, and afatinib. These were all discontinued over 6 months prior to this admission to the hospital. She also received palliative radiation to the right mediastinal mass as well as to several metastatic spine lesions in the cervical and thoracic spine during 2017. Follow-up staging imaging did not show any evidence of radiation pneumonitis or fibrosis. In early 2018, she underwent biopsy of metastatic liver lesions, found to be adenocarcinoma of the lung primary with EGFR exon 19 deletion and T790M mutations. In April 2018, she was started on Osimertinib 80 mg daily, which she tolerated well for approximately four months. A staging CT was completed in June of 2018, which showed response to therapy as evidenced by a decrease in size of the right infrahilar mass, adenopathy, pulmonary nodules, and hepatic metastases. In addition, her previously noted brain metastasis was no longer seen on her brain MRI. In August 2018, she required an admission to an outside facility due to symptoms suggestive of congestive heart failure. She was diuresed and diagnosed with nonischemic cardiomyopathy. Her heart failure remained well compensated on goal-directed medical therapy and diuretics. Other past medical history is notable for hypertension, acid reflux, emphysema, and iron deficiency anemia. She is not prescribed any antiplatelet or anticoagulation agents. She has several allergies to antibiotics and was previously intolerant to subcutaneous heparin.

**Figure 1:**
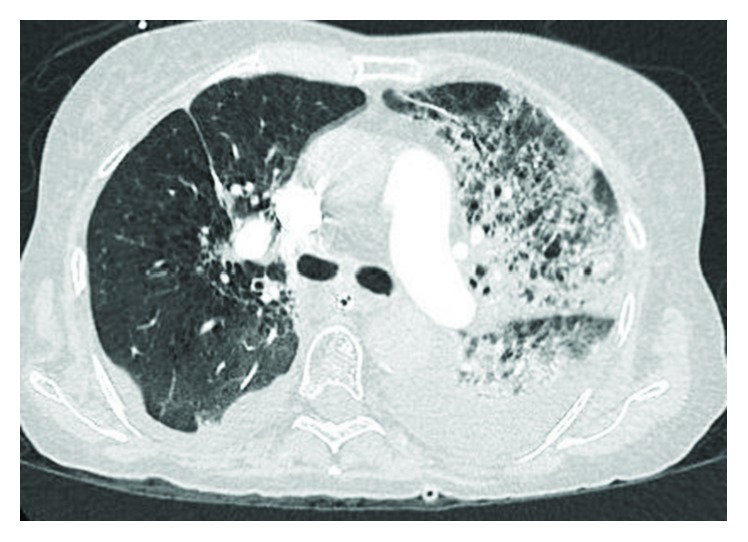
Representative axial CT for PE image of our patient upon admission to the intensive care unit showing dense airspace opacities involving predominantly the left upper lobe and small pleural effusion.

The patient was admitted to our facility in September 2018 due to progressive dyspnea, fatigue, and weakness of several weeks of duration. She was initially managed as hospital-acquired pneumonia in an immunocompromised host with broad-spectrum antimicrobials, aggressive bronchodilators, and intravenous corticosteroids. Within 24 hours of admission, transfer to the intensive care unit was facilitated due to worsening respiratory distress requiring noninvasive positive pressure ventilation which eventually progressed to require mechanical ventilation. At this time, her exam was remarkable for predominant left-sided rhonchi and rales. Laboratory analysis was unremarkable except a severe acute respiratory acidosis of pH 7.18 and PCO_2_ of 54 mmHg. Upon arrival, her WBC count was 7.6 × 10^3^/*μ*L, hemoglobin was 8.5 g/dL, and platelets were 305 × 10^3^/*μ*L, and she had normal coagulation panel and B-type natriuretic peptide (BNP). A CT chest with contrast demonstrated a consolidation in the left lung suspicious for pneumonia and small effusions bilaterally with associated atelectasis (Figure 1). Her antibiotics were continued.

**Figure 2:**
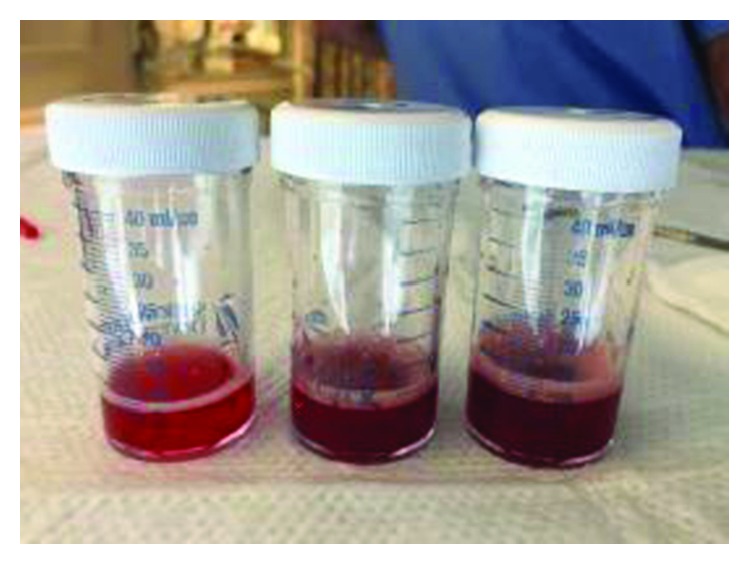
Initial sequential lavages from the patient's lingular segment demonstrating obvious DAH.

**Figure 3:**
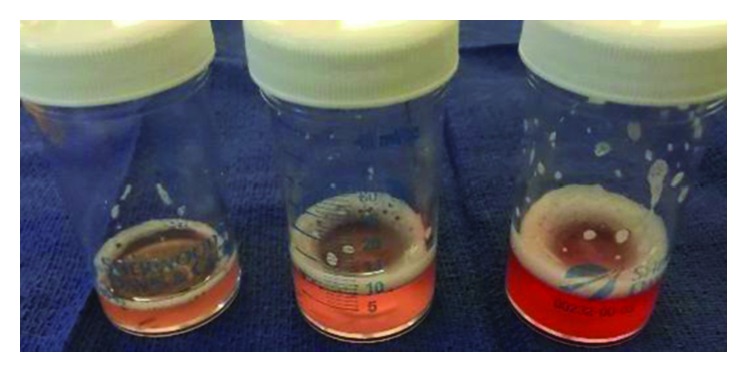
Repeat sequential lavage after 3 days of methylprednisone from lingular segment demonstrating an improvement in her DAH.

Due to the progressive respiratory failure, a bronchoscopy was performed. The bronchoscopy revealed inflamed airways (left>right) and increasingly hemorrhagic return on sequential lavages (Figure 2) from the lingular segment of the left upper lobe. Bronchoalveolar lavage (BAL) RBC counts were increasing significantly in sequential samples (42,000/*μ*L, 190,000/*μ*L, and 230,000/*μ*L). She was diagnosed with DAH with predominant left-sided parenchymal involvement. She was initiated with pulse dose methylprednisone 250 mg IV every 6 hours for 3 days. Her ventilator settings improved significantly after the initiation of pulse dose systemic steroids. After three days of IV steroids, we decided to repeat the bronchoscopy due to continued small amount of blood-tinged tracheal aspirates requiring in-line suction. On repeat bronchoscopy, the airway inflammation had diminished considerably. There was no visible bleeding. We elected to repeat a sequential lavage in the lingular segment, which was dramatically less hemorrhagic (Figure 3). The RBC counts from the sequential lavage were 5,250/*μ*L, 6,750/*μ*L, and 11,500/*μ*L. Microbiology from BAL was unremarkable. The patient was transitioned to oral prednisone 1 mg/kg daily. She was successfully weaned from mechanical ventilation and eventually discharged from the hospital without a need of any supplemental oxygen.

**Figure 4:**
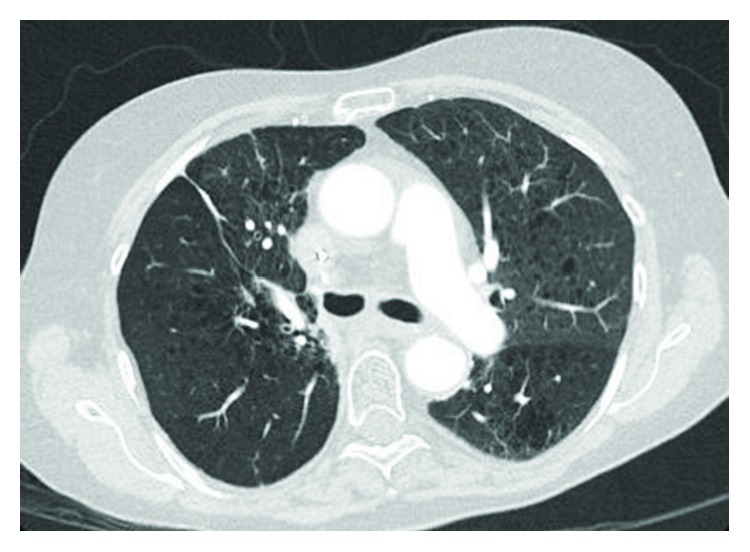
Axial CT chest with contrast approximately 3 months later in an outpatient follow-up, demonstrating a marked improvement of her infiltrate.

A comprehensive standard etiological assessment for DAH was unremarkable including ANCA vasculitis panel and anti-GBM antibodies. Despite her diagnosis of nonischemic cardiomyopathy, the patient was not clinically in decompensated heart failure at any point in her admission. Upon medication review, Osimertinib 80 mg daily was the only new medication the patient had initiated in the prior four months. Osimertinib was discontinued in consultation with medical oncology and was held at her discharge from the hospital. She was discharged on a prolonged prednisone taper. A follow-up CT of the chest demonstrated resolution of the opacities in the left upper lobe (Figure 4).

## 3. Discussion

In recent years, several molecularly defined subsets of non-small cell lung cancer (NSCLC) with specific somatic “driver” mutations, thought to be responsible for the initiation and maintenance of tumor growth, have been identified. Among these mutations, EGFR alterations are the most common, present in about 10% to 15% of patients with NSCLC in the United States. EGFR belongs to the ERBb superfamily of tyrosine kinase receptors, which mediate tumor proliferation, invasion, metastasis, resistance to apoptosis, and angiogenesis. EGFR-TKI (first generation: erlotinib and gefitinib; second generation: afatinib; and most recent: Osimertinib) significantly prolong progression-free survival in patients with advanced NSCLC that contains an activating mutation in EGFR compared with platinum-based chemotherapy doublets [2].

Osimertinib is usually well tolerated with less than 5% of patients developing toxicity from the medication, usually within the first few months. Previously reported pulmonary adverse reactions include pneumonitis including nonspecific interstitial pneumonia [3–6] or other acute interstitial processes [3, 6], fleeting asymptomatic infiltrates on imaging [7, 8], and eosinophilic pneumonia [9]. Previously reported pulmonary toxicities are summarized in Table 1. All of the patients had improvement in their pulmonary complaints after discontinuation of the medication. There seemed to be no typical time frame or age/sex predilection in the previous case reports. Pneumonitis generally appeared sooner than the ILD, within the first month of therapy [4, 5]. ILD, including acute ILD, was a later complication at about 4 months of therapy in the previous case reports [3, 6]. The asymptomatic pulmonary opacities occurred at approximately 8-24 weeks of therapy [7, 8], whereas the eosinophilic pneumonia occurred at 2 weeks of therapy [9]. Some patients were also given steroids which seemed to be an effective adjunct to medication discontinuation in the improvement in respiratory symptoms.

In addition to respiratory adverse reactions, a very small number of patients seem to have developed cardiac adverse drug reactions to Osimertinib (Table 2). Watanabe et al. and Oyakawa et al. described two females, aged 78 and 84, respectively, who developed symptoms of congestive heart failure and dilated cardiomyopathy with no other obvious cause during their course of treatment with Osimertinib [10, 11]. One was 21 days and the other was 34 weeks into treatment [10, 11]. These patients also improved with drug discontinuation and goal-directed medical therapy as well as diuretics. One patient described by Schiefer et al., who was a 61-year-old female, developed a significant QT prolongation after 11 months of treatment with Osimertinib, which quickly normalized after discontinuation of the medication [12].

To our knowledge, this is the first known case reporting diffuse alveolar hemorrhage associated with Osimertinib. Clinicians should be cognizant of this new potential adverse reaction in addition to the already recognized pulmonary and cardiac toxicities of the medication. Urgent evaluation with bronchoscopy and serial bronchoalveolar lavage is critical for quick recognition and diagnosis. Our patient responded very well to discontinuation of the medication and pulse dose steroids with subsequent slow taper. The optimal treatment algorithm is not known but high-dose corticosteroids are likely very effective in bringing prompt resolution to the syndrome. Further investigations into the management of this subset of patients could be undertaken.

With advent of genetic analysis and targeted therapy for lung cancers, there is an increased use of therapy which targets specific genetic mutations. Considering more prevalent use of EGFR-TKI, particularly Osimertinib as front-line therapy for NSCLC, it is highly significant to recognize uncommon adverse reactions. Our case represents the unique side effect of diffuse alveolar hemorrhage leading to severe respiratory failure needing mechanical ventilation with the use of Osimertinib. This paper provides the review of previously reported pulmonary adverse reactions from Osimertinib and highlights the ongoing need for prompt recognition and appropriate management measures to be undertaken during the course of therapy.

## Figures and Tables

**Table 1:** Summary of previously reported cases of pulmonary toxicities of Osimertinib.

Article	Patient age/sex	Onset of symptoms	Symptoms	Diagnosis	Treatment	Outcome
Mamesaya et al. [4]	38 F	31 days	Dyspnea and low-grade fever	Drug-induced interstitial lung disease (ILD)	Withdrawal of medication	Resolution of ILD but progression of malignancy

Matsumoto et al. [5]	75 M	20 days	Generalized weakness and dyspnea	Drug-induced ILD (NSIP)	Methylprednisone 500 mg daily for 3 days, then prednisone 40 mg daily	Resolution of ILD following steroid taper

Yang et al. [3]	8 patients (no specifics)	Average of 5.1 months		ILD and one case of pneumonitis	Not described	3 patients resolved, 2 remained at end of study, and 3 patients deceased

Nie et al. [6]	32 M	4.5 months	Cough and dyspnea	Acute ILD	Dose reduction to 80 mg every other day and dexamethasone 10 mg daily	Improvement in infiltrates and symptoms

Lee et al. [7]	15 patients	24 weeks	None	Asymptomatic pulmonary opacities	None	No adverse events reported

Noonan et al. [8]	4 males (average age 57) and 3 females (average age 43)	8 weeks mean onset	None	Asymptomatic pulmonary opacities, mostly nodules and ground glass opacities	None	All patients had good outcomes

Tachi et al. [9]	77 F	14 days	Fever and hypoxia	Eosinophilic pneumonia due to Osimertinib	Withdrawal of medication	Gradual improvement in her symptoms

**Table 2:** Summary of previously reported cases of cardiac toxicities of Osimertinib.

Article	Patient age/sex	Onset of symptoms	Symptoms	Diagnosis	Treatment	Outcome
Watanabe et al. [10]	78 F	21 days	Facial and lower extremity edema, dyspnea	Congestive heart failure (CHF)	Withdrawal of medication and diuretics	No recurrence of CHF after drug discontinuation

Oyakawa et al. [11]	84 F	34 weeks	Facial edema	Dilated cardiomyopathy/myocarditis	Withdrawal of medication, diuretics, and GDMT	Improved edema, persistently low EF

Schiefer et al. [12]	62 F	11 months	Asymptomatic	QTc prolongation	Withdrawal of medication	Normalization of QT 5 days after discontinuation, patient died of disease progression in 2 months

Yang et al. [3]	6 patients	Unknown	Asymptomatic	QTc prolongation	Dose reduction in 2 patients	Not described
